# Clinical and epidemiological characteristics of influenza virus infection in hospitalized children with acute respiratory infections in Sri Lanka

**DOI:** 10.1371/journal.pone.0272415

**Published:** 2022-09-02

**Authors:** Rukshan A. M. Rafeek, Maduja V. M. Divarathna, Adrian J. Morel, Faseeha Noordeen

**Affiliations:** 1 Department of Microbiology, Faculty of Medicine, University of Peradeniya, Peradeniya, Sri Lanka; 2 Teaching Hospital, Kegalle, Sri Lanka; Carol Davila University of Medicine and Pharmacy: Universitatea de Medicina si Farmacie Carol Davila, ROMANIA

## Abstract

Influenza viruses (Inf-V) are an important cause of acute respiratory infection (ARI) in children. This study was undertaken to describe the clinical and epidemiological characteristics of Inf-V infections in a sample of hospitalized children with ARI. Nasopharyngeal aspirates (NPA) from 500 children between 1 month to 5 years old with symptoms of ARI were collected at the Teaching Hospital Kegalle Sri Lanka From May 2016 to June 2018, NPAs were tested for influenza A (Inf-A) and B (Inf-B) viruses, human respiratory syncytial virus (hRSV), human parainfluenza virus (hPIV) 1–3 using an immunofluorescence assay. The Inf-V were then subtyped using a multiplex RT-PCR. Inf-V were detected in 10.75% (54/502) of the hospitalized children with ARI and in that 5.57% (28/502) were positive for Inf-A and 5.17% (26/502) were positive for Inf-B. Of the 54 Inf-V positive children, 33 were aged between 6 and 20 months. Of the 28 children infected with Inf-A, 15 had uncharacterized lower respiratory infection, 7 had bronchopneumonia and 6 had bronchiolitis. Of the 26 children infected with Inf-B, 11 had uncharacterized lower respiratory infection, 10 had bronchiolitis, and 4 had bronchopneumonia. Inf-B circulated throughout the year with a few peaks, one in June and then in August followed by November to December in 2016 and one in April 2017 and January 2018. Inf-A circulated throughout the year with a major peak in March to April 2017 and July 2018. ARI was more common in boys compared to girls. Majority of the children infected with Inf-V were diagnosed with uncharacterized lower respiratory infection and mild to moderate bronchiolitis. Inf-V infections were prevalent throughout the year in the study area of Sri Lanka with variations in the type of the circulating virus.

## Introduction

Acute respiratory infections (ARIs) are a major public health burden and important causes of paediatric medical visits and admission to hospitals [1]. Human respiratory syncytial virus (hRSV), human parainfluenza virus (hPIV), influenza viruses (Inf-V), adenovirus (AdV), human coronavirus (hCoV) and human metapneumovirus (hMPV) are the most common viral pathogens causing ARI in children <5 years [2]. Of these viruses, Inf-A and B are important causes of ARI ranging from mild flu to severe respiratory illness such as bronchiolitis or complicated pneumonia with substantial morbidity and mortality [3]. Influenza has an estimated annual attack rate of 5 to 10% in adults and 20 to 30% in children with severe respiratory illness in 3 to 5 million cases and half a million deaths globally [4].

Sri Lanka is a part of WHO’s Inf-V surveillance system, which covers only selected hospitals from all nine provinces since the year 2000. The data collected throughout the year from this surveillance represents only a fraction of Inf-V infections occurring in the country as not all of those with influenza will seek a diagnosis and be reported through the surveillance system [5]. According to this surveillance report Inf-V circulate throughout the year with peaks in April-June and November to February [5]. Despite the increase in global influenza surveillance and improved understanding of Inf-V, longitudinal data on seasonality, epidemiology and estimates of influenza-associated disease burden are limited in tropical countries like Sri Lanka [5, 6]. However, it appears that the distribution and mortality from influenza in tropical countries are comparable to those seen in temperate high-income countries [7]. This study describes the epidemiological, clinical characteristics and seasonality of Inf-V infections in a sample of children hospitalised for ARI in Teaching Hospital, Kegalle (THK), Sri Lanka.

## Materials and methods

### Study design and setting

This was a descriptive cross-sectional study conducted at THK, Sri Lanka from May 2016 to July 2018. THK serves as the tertiary referral centre for Kegalle District providing healthcare services to nearly 1 million people in the district. According to the 2012 Census the district has a population of 837,179 which is approximately 4.0% of the total population of Sri Lanka and 204, 261 are in the 0–14 years age group. Kegalle district geographically belongs to the north-eastern wet zone and it receives heavy rain as well as bright sun shine throughout the year with annual rainfall of 2500–3000 mm and average temperature of 27.9 °C.

### Study participants and clinical definitions

Children between 1 month to 5 years of age admitted for an influenza-like illness (ILI) with fever of ~38°C, cough and onset of ILI within the past 4 days were screened by the consultant paediatrician. All those who did not meet the case definition or not willing to participate were excluded from the study. Any child above the age of 5 years and below 1 month despite being clinically diagnosed with ARI and any child with respiratory symptoms suggesting anything other than ARI were also excluded from the study. Demographic and clinical data were collected using a standard questionnaire.

### Ethics statement

This study was approved by the Ethics Review Committee of the Faculty of Medicine, University of Peradeniya, Sri Lanka in accordance with the guidelines for the protection of human subjects (2016/EC/91). Written informed consent was obtained from parents or legal guardians of children before enrolling them to the study.

### Collection of climatic data

The climatic data including temperature, humidity, rainfall and number of rainy days were collected from World Weather Online Application Program Interface (WWO-API) to determine the influence of these factors on the distribution of Inf-A and -B infections to predict future outbreaks with peak Inf-V transmission in a year.

### Clinical samples and virus detection

Nasopharyngeal aspirates (NPA) were collected by trained medical personnel within 24 hours of admission and were transported to the Virology Laboratory of the Faculty of Medicine, University of Peradeniya. NPAs were processed for antigen detection using an indirect immunofluorescence assay—a screening test followed by a direct immunofluorescence assay to detect eight common respiratory viruses (Inf-A and B, hRSV, hPIV 1, 2 and 3, hMPV and hAdV) (Diagnostic Hybrids, Inc. D3*Ultra™* DFA respiratory virus screening & id kit, USA) following the manufacturer’s instructions. The slides were examined using a fluorescence microscope (UV Epi fluorescence microscope, Leads, Germany) with magnifications of 200X to 400X. The nasopharyngeal epithelial cells with intracellular nuclear and/or cytoplasmic granular apple green fluorescence were considered as positive for Inf-A and -B antigen. Cells that were stained red with Evan’s blue counter stain without any fluorescence were considered as negative (S1 Fig in S1 File).

### Laboratory diagnosis based on viral test results

At the time of the sample collection THK did not have facility to perform antigen-based test or molecular testing for respiratory virus diagnosis. Therefore, this study was conducted in agreement with reporting of laboratory diagnosis of viral causes of ARI in children by DFA within 24 hours of sample collection as part of standard clinical practice. Positive virus identification by DFA was reported to clinicians for further care for children and to avoid unnecessary antibiotic usage.

### RT-PCR to characterise Inf-A types

All specimens positive for Inf-A by DFA were sub-typed using RT-PCR. A previously published [8] multiplex PCR for sub-typing of Inf-A H1N1 and H3N2 and a separate PCR (WHO protocol) for Inf-A H1N1pdm09 were used for characterizing Inf-A. Briefly, viral RNA was extracted using QIAmp Viral RNA mini kit (QIAGEN^™^, Germany) from the NPA samples for the synthesis of cDNA. Prior to reverse transcription (RT), the viral RNA was preheated at 65°C for 5 minutes with the forward primer, Unit 12M/Oligo(dT)12-18 Primer (20mM) (Promega). The RT reaction mixture contained preheated RNA extract, 4μL of 50 RT-MMLV buffer, 10 mM of DTT, 20U of RT-MMLV enzyme (Promega), 1 μL of reverse primer (20mM) and RNAase free water to make a total reaction volume of 25μL. RT mixture was then incubated at 42°C for 60 minutes. Multiplex PCR targeting two subtypes of Inf-A H1N1 and H3N2 was performed using primers targeting M and H genes of the Inf-A (S1 Table in S1 File).

### Data analysis

Data were double-checked and entered into a spreadsheet prepared in Microsoft^®^ Excel 2013 and statistical analysis was done using the statistical package Minitab version 17.0 (Pennsylvania State University). General data were shown as percentages or means with 95% confidence intervals (CI). Categorical data was analysed using either Chi-Square (χ2 test) or Fisher’s Exact Test as appropriate. *p* value of <0.05 was considered statistically significant for all tests. The correlation between the monthly distribution of Inf-A and–B associated ARI and climatic factors including mean monthly atmospheric temperature (ºC), mean monthly relative humidity (%), mean monthly rainfall (mm), and mean rainy days in a month (n) was done using Spearman’s correlation coefficient. Multivariable logistic regression analysis was performed to determine the risk factors associated with Inf-A and -B infections. Odd ratios (ORs) were calculated for common risk factors (categorical variable) of ARI described in Table 2.

## Results

### Demographic and clinical characteristics of the study population

This study enrolled 500 children between 1 month to 5 years of age with ARI from May 2016 to July 2018. The demographic and clinical characteristics of the study population were given in Table 1. Of the 500 children recruited 304 (61.3%) were males and 196 (38.7%) were females where the number of boys with ARI was significantly higher than that of girls (χ2 = 8.174, df = 15.63, 1, *p* = 0.03). Of recruited children, 244 had at least one of the seven viral infection including Inf-V, hRSV and HPIV 1–3. Of the 500 children with ARI, 54 were positive for Inf-V (10.8%), of whom 28 (5.6%) and 26 (5.2%) were positive for Inf-A and Inf-B, respectively. Rest of the 190 children were positive for other common respiratory viruses including hRSV and HPIV 1–3. However, there is no significant difference between boys and girls (χ2 = 0.3045, df = 0.04, 1 *p* = 0.76). Of the 28 Inf-A positive children, 10 had pandemic H1N1pdm09, 18 had seasonal H3N2 and none of them were positive for seasonal H1N1. There were no significant differences in the incidence (*p* = 0.58) and severity in children less than 5 years with pandemic H1N1pdm09 and seasonal H3N2 infections (S2 Table in S1 File). None of the children infected with pandemic H1N1pdm09 and seasonal H3N2 required high dependency care or any additional medical care.

**Table 1:** Demographic and clinical characteristics of children with Inf-V infections.

Characteristics / Virus type	Influenza A (n = 28)	Influenza B (n = 26)	Other viruses—hRSV and hPIV 1–3 (n = 190)	Virus Negative (n = 256)	*χ2* (Chi-square, df)	*p*-value
**Demographic characteristics**						
Mean Age ± SD (Months)	18.2 ± 19.6	14.7 ± 10.5	10.82 ± 10.11	14.2 ± 12.6	-	0.567
Mean fever days	3.18 ± 1.09	2.42 ± 0.99	2.69 ± 1.1	4.09 ± 1.46	-	0.646
Male: Female Female = ref	15:13	15:11	117:72	152:106	0.23 (0.05, 1)	0.647
Residential area Urban: Rural Urban = ref	9:19	12:14	79:111	118:140	0.48 (0.23, 1)	0.371
**Clinical characteristics**						
Fever	27	21	164	201	0.60 (0.36, 1)	0.582
Cough	26	25	185	235	0.16 (0.02, 1)	0.036
Cold	19	22	164	177	0.44 (0.19, 1)	0.730
Sore throat	11	14	5	4	**9.01 (81.35, 1)**	<0.001*
Runny nose	26	24	164	225	0.27 (0.07, 1)	0.828
Shortness of breath (SOB)	0	9	27	39	0.24 (0.05, 1)	0.838
Difficulty in breathing	9	15	108	123	0.26 (0.06, 1)	0.894
Wheezing	0	2	8	17	0.76 (0.58, 1)	0.751
Headache	12	12	11	23	**5.23 (27.37, 1)**	<0.001*
Vomiting	7	2	35	48	0.28 (0.07, 1)	0.850
Dyspnoea	7	6	62	43	1.05 (1.11, 1)	0.346
Conjunctivitis	2	0	4	23	0.33 (0.10, 1)	0.398
Tachypnoea	9	3	57	39	1.06 (1.14, 1)	0.326
Nasal block	0	3	12	16	0.17 (0.02, 1)	>0.999
Chills	15	16	20	21	**6.01 (36.18, 1)**	<0.001*
Diarrhoea	6	2	14	8	**3.25 (10.61, 1)**	0.003*
Fatigue	12	16	5	4	**8.07 (65.22, 1)**	<0.001*
Body aches	13	15	7	4	**8.87 78.76, 1**	<0.001*

Demographic characteristics including age, fever days, gender, residential area and common clinical characteristics of ARI were compared between children infected with Inf-V and children negative for any of the viruses. *p* values indicate the significant difference in demographic and clinical characteristics Inf-V infection in hospitalised children (*statistical significance at p<0.05).

**Fig 1:**
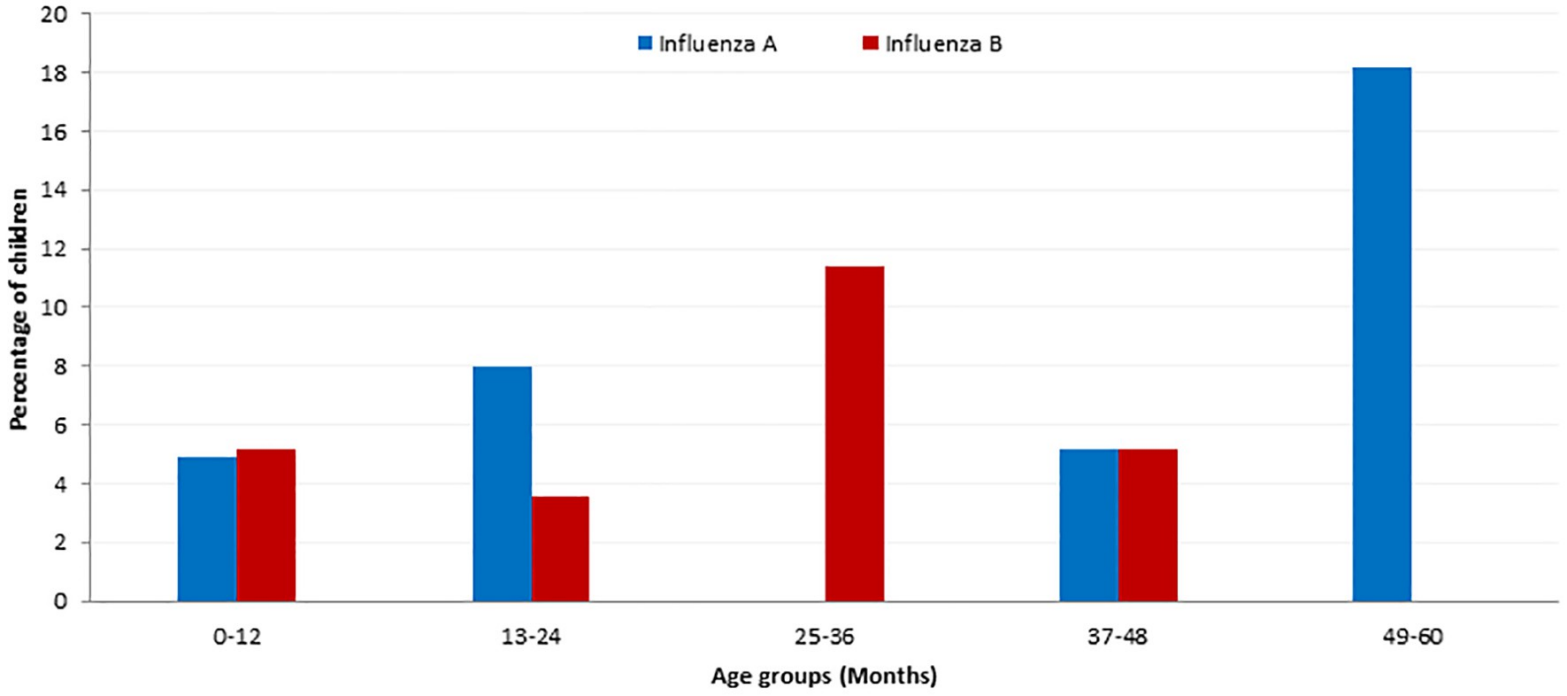
Age distributions of Inf-V infections in children with ARI. The median age of children with ARI was 13 months (Mean ± SD; 13.166 ± 12.394). Out of the 54 Inf-V infections, 33 (61.1%) were less than 12 months old. The rate of Inf-V infection increased with the increase in age in children less than 5 years old.

The mean age of Inf-V infected children was 11.38±15.75 months and the incidence of Inf-V infection was higher in children less than 1 year (Fig 1). Children from rural areas were infected with the Inf-V more frequently including Inf-A (urban 32.14%; rural 67.85%) and Inf-B (urban 46.2%; rural 53.8%). The mean fever days of children with Inf-V infection was 3.18±1.09 days (Table 1).

**Fig 2:**
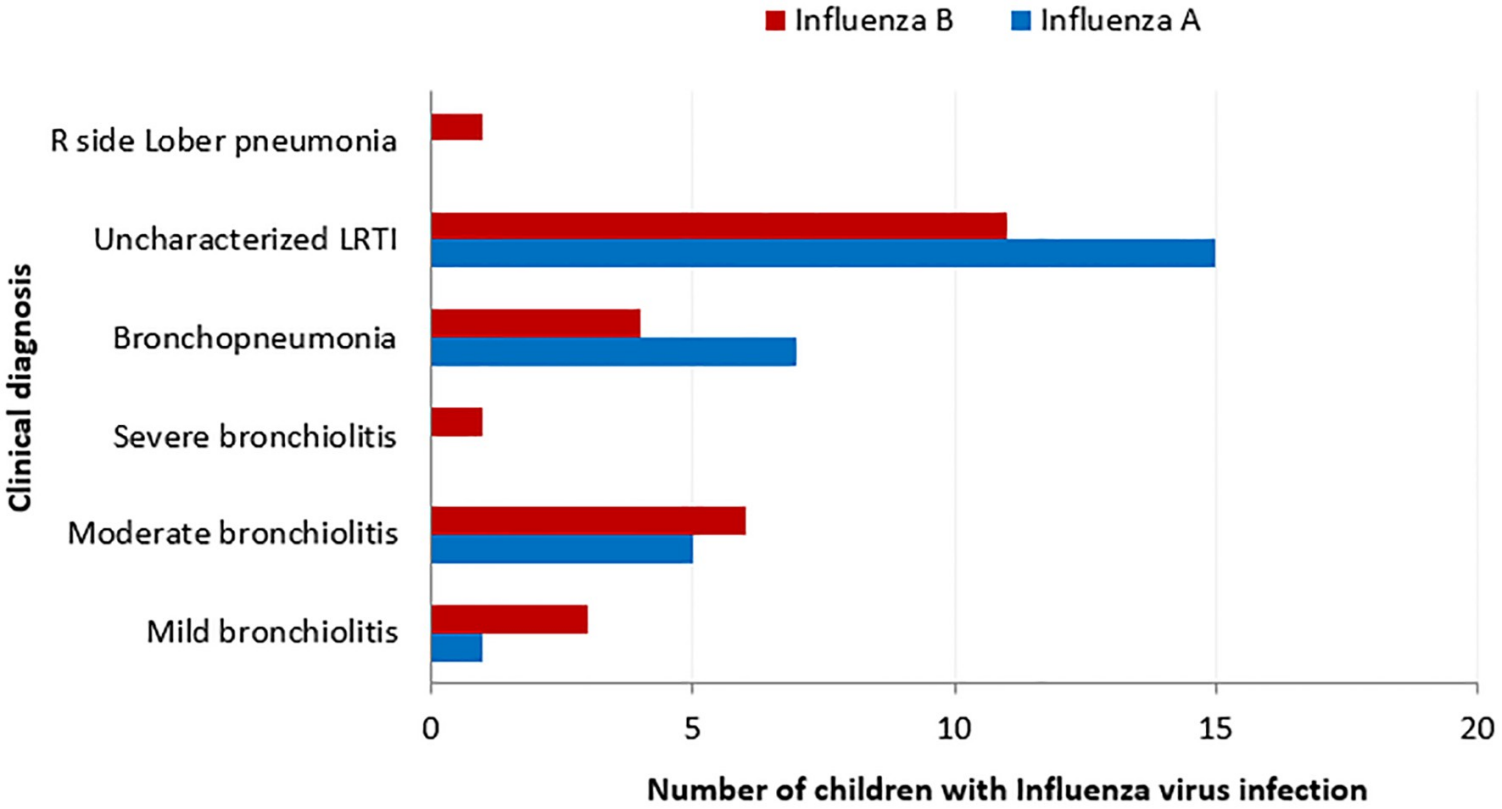
Clinical diagnosis of Inf-V associated ARI in children. Bronchiolitis is a common LRI and is due to the inflammation of the bronchioles it can be either mild, moderate or severe; Bronchopneumonia is pneumonia due to inflammation in the alveoli affecting bronchi and bronchioles; Uncharacterized lower respiratory infection is lower respiratory infection, which is not bronchiolitis or bronchopneumonia; Right side lobar pneumonia is inflammation in the lobes in the right lung.

Fever (96.42%), cough (96.15%), cold (84.61%) and runny nose (92.85%) were common signs and symptoms observed in children infected with Inf-V on admission (Table 1). Binary logistic regression analysis revealed positive associations of Inf-V with fever (*p* = 0.31, OR 1.1; 95%CI, 0.7 to 1.7), cough (*p* = 0.43, OR 1.0; 95%CI, 0.6 to 1.5), cold (*p* = 0.32, OR 1.107; 95%CI, 0.69 to 1.71) and runny nose in children. Shortness of breath (SOB) and difficulty in breathing were observed only in children infected with Inf-B. Dyspnoea, tachypnoea and diarrhoea were more common in children infected with Inf-A. Pain-related symptoms including body aches, fatigue, chills, headache and sore throat were frequently observed in children infected with Inf-A and B (Table 1). Wheezing was noted in 3.7% of the children and gastrointestinal symptoms were noted in children infected with the H1N1pdm09 virus (40%) (S2 Table in S1 File). Binary logistic regression was performed to identify the association between commonly detected respiratory clinical characteristics, including uncharacterized lower respiratory infection, moderate bronchiolitis, mild bronchiolitis, bronchopneumonia, severe bronchiolitis and right sided lobular pneumonia and the infecting Inf-V. As shown in Fig 2, Inf-A (*p*< 0.001, OR = 4.42; 95%CI, 1.92 to 10.13) and Inf-B (*p*< 0.001, OR = 17.80; 95%CI, 3.68 to 86.09) were associated with uncharacterized lower respiratory infection. We compared the clinical features of patients with Inf-A infection (n = 28) to those with Inf-B infection (n = 26). Presence of typical respiratory symptoms (fever, cough and sore throat) was not significantly different between Inf-A and B infections. However, children with Inf-B infection were more likely to have nasal block, wheezing, fatigue and SOB than those with Inf-A infection (*p* = 0.045).

To understand the association between the Inf-V and four commonly noted clinical characteristics: uncharacterized LRI, moderate bronchiolitis, mild bronchiolitis and bronchopneumonia, a binary logistic regression was performed. The Inf-V was associated with 48.14% uncharacterized LRI, 20.37% of bronchopneumonia and moderate bronchiolitis, 7.14% mild bronchiolitis and 1.85% of severe bronchiolitis and R side lobar pneumonia in children.

### Detection of respiratory viruses and co-infections

Of the 54 Inf-V infected children, 13 had respiratory viral co-infections. Except for a child who had triple virus infection, all others had dual infections. Four co-infections were between Inf-A and hRSV, one between Inf-A and hPIV-3, one between Inf-A and Inf-B. Five co-infections were between Inf-B and hRSV and one between Inf-A, Inf-B and hRSV. However, the child with the triple co-infection with Inf-A, Inf-B and hRSV did not have severe disease and the chest X-ray was normal. A child with the co-infection with Inf-A and hPIV-3 showed bilateral inflammation in the chest X-ray. Co-infection was observed commonly in boys (66.05%, 8/13) and co-infection was more common in children between 2–3 years (6.02%) than in children above 4 years (1.42%).

### Risk factors for acquiring Inf-V associated ARI

Male sex, exposure to cigarette smoke, family history of asthma and allergic reaction to pollen/dust were associated with ARI, none of them were significant risk factors for acquiring any of the Inf-V in the current study. Day care attendance and Trisomy 21 were identified as significant risk factors for acquiring Inf-A infections (*p* = 0.004, OR = 17.8; 95%CI, 2.8 to 114.0 and *p* = 0.042, OR = 18.5; 95%CI, 1.1 to 304.7, respectively). Although there was more than 1.25-fold increase in the odds for acquiring Inf-A infections with indoor air pollution, crowded living conditions (more than 4 people in a house) and congenital cardiac and respiratory anomalies and this increase was not statistically significant (*p =* 0.33, 0.52 and 0.51, respectively). Indoor air pollution, travel history in the past 14 days and exposures to animals increased the association with Inf-B infections but this association was also not statistically significant (Table 2). These inferences were made based on the binary logistic regression analysis was performed to determine the risk factors for acquiring Inf-V infection and hospitalization.

**Table 2:** Risk factors for acquiring Inf-V associated ARI in the study sample.

Characteristics	Inf-A (n = 28)		Inf-B (n = 26)	
	Odds ratio (95%CI)	*p* Value	Odds ratio (95%CI)	*p* Value
Gender (male)	0.7 (0.6–6.1)	0.46	0.46 (0.08–1.5)	0.77
Day care attendance	17.8 (2.4–131.5)	**0.004** *	0 (-)	0
Outdoor air pollution	1.000 (0.5–1.8)	0.10	0 (-)	0
Indoor air pollution	1.6 (0.05–5.6)	0.33	4.26 (1.5–7.2)	0.05
Crowded living conditions (<4 people)	1.3 (0.5–1.7)	0.52	0.69 (0.4–1.3)	0.38
Exposure to cigarette smoke	0.2 (0.01–2.0)	0.16	0 (-)	0
Congenital cardiac and respiratory anomalies	2.0 (0.3–18.1)	0.51	0 (-)	0
Family history of respiratory disease/asthma	0.7 (0.4–1.3)	0.65	0 (-)	0
Trisomy 21 child	18.5 (1.1 to 304.7)	**0.04** *	0 (-)	0
Travel in past 14 days	0 (-)	0	1.14 (0.4–1.7)	0.90
Allergic reaction to pollen/dust	0 (-)	0	0 (-)	0
Animal exposure (Dogs, cats and birds)	0.5 (1.0 to 4.1)	0.70	1.15 (0.4–1.5)	0.74

*Statistical significance at p<0.05.

### Influence of climatic factors on Inf-V associated ARI

**Fig 3:**
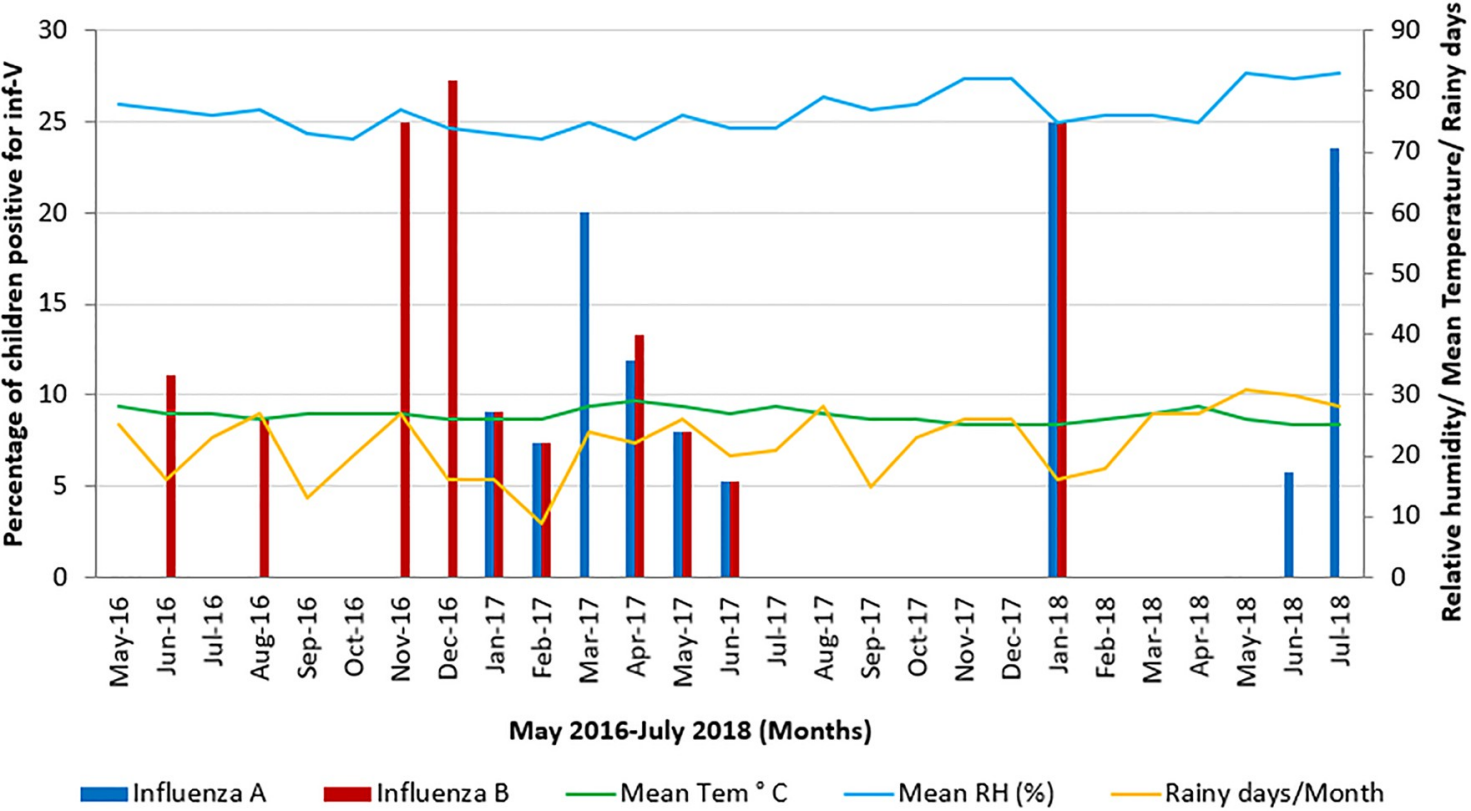
Monthly distribution of Inf-V in children with ARI. Monthly distribution of the Inf-V infection in children from May 2016 to July 2018. The months of November to December 2016, March 2017 and June to July 2018 had children with high percentage of Inf-V infection on admission to Teaching Hospital, Kegalle Sri Lanka.

In the study area, children were infected with Inf-V throughout the year with two peaks from November 2016 to May in 2017 and June to July in 2018. The highest rate of Inf-A infection was detected in March to April 2017 (20% and 11.9%), January 2018 (25%) and July 2018 (23.5%); the highest rate of Inf-B infection was detected in November and December 2016 and January 2018 (25%, 25.27%, and 25%, respectively). No cases with Inf-V infections were detected in July to December 2017 and February to May 2018, respectively (Fig 3). The effect of climatic factors on influenza was evaluated on a monthly basis (Fig 3).

The Spearman’s rank and correlations showed a positive correlation between Inf-V infection and mean monthly atmospheric temperature (r^2^ = 0.152) and number of rainy days in a month (r^2^ = 0.087) and an indirect correlation with mean monthly relative humidity (r^2^ = -0.117%) and mean monthly rainfall (r^2^ = -0.094) (S3 Table in S1 File).

## Discussion

ARI remains the second leading cause of death worldwide in children younger than 5 years of age [1, 4]. According to the Ministry of Health Reports of Sri Lanka, approximately one-fifth of ARI cases were occurring in children <5 years of age [9]. However, Sri Lanka has limited epidemiological information about the diversity of viruses causing ARI and the impact of influenza in childhood ARI [5]. Here we report clinical, epidemiological characteristics of Inf-V associated ARI in a selected sample of hospitalized children in Sri Lanka. Using antigen detection by DFA, we demonstrated that 10.8% of the children had Inf-V infection and this detection rate is comparable with the study by Shapiro et al. (2017) from Sri Lanka, although that study only enrolled outpatients [10]. Distribution of Inf-V infection in our study is consistent with the reports from few other studies conducted in Sri Lanka and south Asian countries [10–13], but lower than that reported in countries like Hong Kong and China [3, 14], reinforcing that epidemiology of influenza is highly heterogenic in different countries. However, the prevalence of Inf-Vinfection in our study was also higher than that reported in two previous studies from Sri Lanka [11, 15] and reinforcing that epidemiology of influenza is also highly heterogenic in different areas within a country. Moreover, this discrepancy may be due to the difference in the study duration, nature of the study population and nature of the respiratory specimen tested and methods used to test.

In the present study, 61.11% of the children younger than 1 year of age were likely to have Inf-V associated ARI, however, the rate of Inf-V infection increased with increase in age. Previous studies report high rates of influenza-associated hospitalisation in those aged <5 years, especially in infants in their first year of life [16, 17]. Although we had high number of ARIs in children less than 12 months of age, the percentage of Inf-V positive cases were less in this age category. Viral ARIs are more common in males [10, 16] and our data also showed a slight male predominance (55.6%) among the virus positive children but the overall positive detection rate was not significantly different between boys and girls. The discrepancy in the Inf-V infection between males and females might be due to susceptibility and presence of predisposing risk factors [18, 19].

We also compared signs and symptoms of children with Inf-V associated ARI and our study showed that cough, fever and difficulty in breathing were associated with Inf-V positivity, corroborating with findings from previous studies [2, 10]. The presenting symptoms of Inf-V infection in children do not differ greatly between different geographical regions. In agreement with previous studies [2, 10], the clinical symptoms associated with Inf-B infections were similar to Inf-A infection in the current study too. Fever and cough are the most common symptoms in children with Inf-V infection [2, 9, 10] and in the current study, similar number of Inf-A and Inf-B infected children presented with a temperature ≥38°C. However, Inf-A infected children are more likely to have fever >38°C than Inf-B infected children [10, 11, 13, 14]. Up to 15% of the children with Inf-V infection presented with gastrointestinal symptoms such as vomiting and diarrhoea, however, previous studies [2, 10] report gastrointestinal symptoms in more than 50% of the children. SOB, nasal block, wheezing and nasal congestion were mostly associated with Inf-B than Inf-A infections (Table 1). In agreement with previous findings [10, 20], the clinical manifestations of Inf-V infections in the present study were mild to moderate in many children even though very young children are a risk group for influenza associated ARI. None of the children infected with Inf-V or other respiratory virus infection required an intensive care.

Our results show that viral co-infection with Inf-V is more frequent in children less than 36 months old. The rate of Inf-V co-infection with other respiratory viruses is 30%, as previously reported, hRSV was the more frequently co-infecting virus followed by hPIV-3 [2, 10]. Children co-infected with Inf-V and hRSV were clinically diagnosed with bronchiolitis, however, children co-infected with Inf-V and hPIV-3 were clinically diagnosed with uncharacterized lower respiratory infection. In this study, children with Inf-V mono infection were diagnosed with uncharacterized lower respiratory infection or bronchopneumonia. Since hRSV is the commonest cause of bronchiolitis, which may be overshadowed by uncharacterized lower respiratory infection in children with co-infection of hRSV and Inf-V (Fig 2). Like previous findings we found no differences in the clinical severity between children with Inf-V mono-infection and Inf-V co-infections with hRSV or hPIV [21]. We observed that co-infection rates were lower in older children (>24 months), despite this group being prone to high exposure to the virus through increased participation in shared childcare groups. Previous studies show male sex [16, 18], outdoor air pollution due to the intense motor traffic and urbanization [22, 23], exposure to cigarette smoke [24, 25], family history of respiratory disease/asthma [26, 27], travel in the past 14 days [28], allergic reaction to pollen/dust [29] and exposures to animals [30] are significant risk factors for Inf-V infection in children. However, our study did not show any significant differences in those risk factors in children with Inf-V infection and respiratory viral co-infections. However, as noted by other studies, this study also showed day care attendance [31, 32], indoor air pollution [22, 33], crowded living conditions (<4 people) [34, 35], congenital cardiac and respiratory anomalies [36], Trisomy 21 [37, 38] as common risk factors for acquiring Inf-V infection in children. Identifying the common risk factors associated with Inf-V infection and respiratory viral co-infections will help parents / guardians to practice preventive measures to minimize the spread of these infections.

Although studies from temperate and sub-tropical countries show an association, between distribution of Inf-V infection and climatic conditions we did not find any association between climatic conditions and influenza incidence in the study except for atmospheric temperature, which is statistically not significant. According to the Ministry of Health of Sri Lanka, influenza occurs in two peaks annually, with the larger peak occurring in May to July and the second peak occurring in November to January [5]. Our study also observed a similar pattern of Inf-V infection in children less than 5 years old (Fig 3). In the country, influenza season from 2016 to 2018 was characterised by co-circulation of two Inf-V types (Inf-A and Inf-B) and two Inf-A subtypes (Inf-A H3N2, Inf-A H1N1pdm09). In accordance with the national influenza surveillance data and studies from other countries [7, 39], the current study also reports co-circulation of two Inf-A subtypes (Inf-A H3N2, Inf-A H1N1pdm09) and Inf-B during the study period, from 2016 to 2018.

Based on the current study on seasonality, Inf-V circulated throughout the year in the study area and was presented in a bi-modal curve with two peaks coinciding both monsoons and this pattern is in agreement with data from other tropical Asian countries like Singapore [7]. In contrast, the virus activity correlates with rainfall in Thailand [40]. Study by Shapiro et al. showed that Inf-A and respiratory viral activity was peaked in February–June of 2013 and 2014 in the Southern province of Sri Lanka [10]. According to the WHO’s FluNet report from 2010 to 2015 and national surveillance data from 2000–2008 and 2011–2014, the major influenza peak was observed in October–December with a lesser peak in January–March in Sri Lanka [41]. Taken together, the seasonality of respiratory viruses including Inf-V varies widely in tropical and subtropical countries and different climatic zones within a country due to variations in temperature, humidity, and rainfall [42]. The current study was done only for 26 months in a selected area of Sri Lanka and data for several years are needed to draw a better conclusion on the seasonality of Inf-V activity in the country.

Rapid and accurate identification of the viruses causing ARIs is important in order to initiate appropriate antiviral therapy and to prevent the overuse of antibiotics, nosocomial transmission and to minimize the protracted hospital stay [43]. Moreover, there is no national vaccination program for influenza in Sri Lanka and only less than 1% of the Sri Lankan population receives influenza vaccination. It is a challenge to implement the vaccination due to two large peaks of Inf-V circulation separated by several months in Sri Lanka [41, 44, 45]. Molecular techniques with higher sensitivity and rapidity play a critical role in the early identification of respiratory viruses particularly during epidemics [46]. The virus-detection rate varies depending on the specimen type, the method used and the time when the sampling is done.

This study has enrolled only hospitalized children aged between 1 month and 5 years from a single centre and thus the results cannot be extrapolated to the overall paediatric population in the country. However, the current study was able to bring out important aspects of Inf-V infections in children in Sri Lanka laying a foundation for bigger longitudinal studies. Furthermore our study did not performed any molecular characterization for influenza B strains. The rate of Inf-B associated ARI in this study reinforce the importance of screening and typing of Inf-B.

## Conclusion

In conclusion, 10.75% of the children had Inf-V associated ARI, of this, 5.6% and 5.2% were Inf-A and Inf-B associated ARI, respectively. Majority of the children infected were less than 18 months old and the rate of Inf-V infection has decreased with increasing age. The frequent symptoms of children infected with Inf-V were cough, fever and difficulty in breathing. During the study period, Inf-V infection peaked in June, August and in November to December in 2016 and 2017. Predominantly circulated Inf-A subtype during the study period was Inf-A H3N2 followed by Inf-A H1N1pdm09. There was no correlation noted between climatic factors and incidence of Inf-V infections during the study period. As the aetiology of ARI cannot be differentiate by the clinical features only, this study shows the importance of rapid laboratory diagnosis of Inf-V infection in hospitalized children with ARI. The use of DFA in combination with RT-PCR allow identification and characterization of Inf-V infections in resource-limited countries for the improvement of diagnosis algorithms for better clinical management of ARI.

## Supporting information

S1 File (DOCX)
