# Supplementary material for: Clinical and epidemiological characteristics of influenza virus infection in hospitalized children with acute respiratory infections in Sri Lanka
Source: PLoS One. 2022 Sep 2;17(9):e0272415. doi: 10.1371/journal.pone.0272415 (PMC9439189; doi:10.1371/journal.pone.0272415)
Supplement: S1 File — (DOCX) [file pone.0272415.s001.docx]

Supporting Information

**Clinical and epidemiological characteristics and seasonality of influenza virus associated acute respiratory infections in a selected sample of hospitalized children in Sri Lanka**

Rukshan AM Rafeek^1^, Maduja VM Divarathna^1^, Adrian J Morel^2^,Faseeha Noordeen^1^*

^1^Department of Microbiology, Faculty of Medicine, University of Peradeniya, Sri Lanka

^2^Teaching Hospital, Kegalle, Sri Lanka

*Corresponding Author

E mail: [faseeha.noordeen@med.pdn.ac.lk](mailto:faseeha.noordeen@med.pdn.ac.lk); [faseeha.noordeen12@gmail.com](mailto:faseeha.noordeen12@gmail.com)

Contact Details: +94772293301


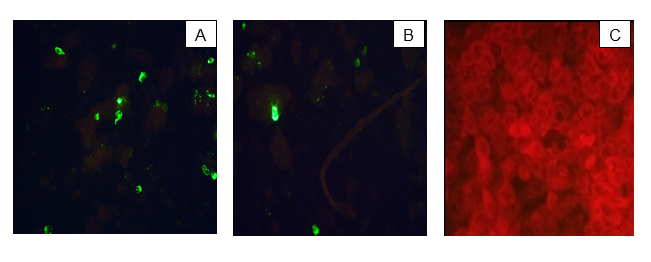
S1 Fig. Florescence microscopic appearance of infected epithelial cells from NPAs of children expressing viral antigens in apple green in colour. A typical bright fluorescence in the cytoplasm with a dark nucleus of the epithelial cells in DFA was considered positive. However, in Inf-A infection both nuclear and cytoplasmic fluorescence was noted. The viral antigen positive cells were predominantly ciliated epithelial cells. A - Inf-A, B- Inf-B and C - negative control (40 × 10× magnification).

S1 Table. Primers used for sub typing of Inf-A H1N1, H3N2 and H1N1 pdm09.

| **Primers** | **Nucleotide sequences of primers** | **Target genes** |
| --- | --- | --- |
| Unit 12 M (Oligo(dT)_12-18_ Primer) | AgCRAAAgCAgg | HA, M1 & M2 |
| Forward primer M-F2 | AgTgAgCgACTgCAgCgT | M (common type A) 358 213–233 |
| Reverse primer M-R2 | TAgCY*TTAgCY*gTR*gTgCTggC |  |
| Forward primer AH1-PD-F6 | ACAgTgACACACTCTgTCgTCAA | H (A/H1N1) |
| Reverse primer AH1-PD-R2 | ACACTCTCCTATTgTgACTggg |  |
| Forward primer AH3-PD-F1-2 | TgggAgACCCTCATTgTgATg | H (A/H3N2) |
| Reverse primer AH3-PD-R1 | TTgggAATgCTTCCATTTgg |  |
| Forward primer HKU-SWF | GAGCTCAGTGTCATCATTTGAA | H (A/H1N1) pdm09 |
| Reverse primer HKU-SWR | TGCTGAGCTTTGGGTATGAA |  |

* T, C, A, G - Degeneration site level 4;

Y - Pyrimidine base (T or C), level 2;

R - Purine base (A or G), degeneration site level 2

**S2 Table. Demographic and clinical characteristics of children with Inf-A H1N1pdm09 and H3N2 infections.**

| **Characteristics / Virus type** | **Influenza A** **H1N1pdm09 (n=10)** | **Influenza A** **H3N2**  **(n=18)** |
| --- | --- | --- |
| **Demographic characteristics** |  | |
| Mean Age ± SD (Months) | 15.5 ± 12.3 | 19.7 ± 22.8 |
| Mean fever days | 3.6 ± 1.075 | 2.94 ± 1.06 |
| Male: Female  Female = ref | 6:4 | 9:9 |
| Residential area  Urban: Rural  Urban = ref | 2:8 | 7:11 |
| **Clinical characteristics** |  | |
| Fever | 10 | 17 |
| Cough | 10 | 16 |
| Cold | 5 | 14 |
| Sore throat | 3 | 8 |
| Runny nose | 9 | 17 |
| Shortness of breath (SOB) | 0 | 0 |
| Difficulty in breathing | 3 | 6 |
| Wheezing | 0 | 0 |
| Headache | 3 | 9 |
| Vomiting | 4 | 3 |
| Dyspnoea | 3 | 4 |
| Conjunctivitis | 0 | 2 |
| Tachypnoea | 5 | 4 |
| Nasal block | 0 | 0 |
| Chills | 3 | 12 |
| Diarrhoea | 4 | 2 |
| Fatigue | 1 | 11 |
| Body aches | 5 | 8 |

Demographic characteristics including age, fever days, gender, residential area and common clinical characteristics of ARI were compared between children infected with Inf-V and children negative for any of the viruses. *p* value indicates the significant difference in demographic and clinical characteristics Inf-V infection in hospitalised children. *Significantly different (* p=<0.05)

S3 Table. Correlation between climatic factors and influenza virus associated ARIs in the study sample.

| **Climatic factors** | **Duration of study in months** | **Spearman’s Correlation coefficient** | ***p* value** |
| --- | --- | --- | --- |
| Mean monthly atmospheric temperature (ºC) | 27 | 0.152 | 0.535 |
| Mean monthly relative humidity (%) | 27 | -0.117 | 0.634 |
| Mean monthly rainfall (mm) | 27 | -0.094 | 0.701 |
| Mean of rainy days in a month (n) | 27 | 0.087 | 0.723 |
